# A Prospective Longitudinal Cohort to Investigate the Effects of Early Life Giardiasis on Growth and All Cause Diarrhea

**DOI:** 10.1093/cid/ciw391

**Published:** 2016-06-16

**Authors:** Jeffrey R. Donowitz, Masud Alam, Mamun Kabir, Jennie Z. Ma, Forida Nazib, James A. Platts-Mills, Luther A. Bartelt, Rashidul Haque, William A. Petri

**Affiliations:** 1 Division of Pediatric Infectious Diseases, Children's Hospital of Richmond at Virginia Commonwealth University; 2 Parasitology Laboratory, International Centre for Diarrhoeal Disease Research, Bangladesh, Dhaka; 3 Department of Public Health Sciences, University of Virginia, Charlottesville; 4 Department of Medicine and Vaccine Testing Center, The University of Vermont College of Medicine, Burlington; 5 Division of Infectious Diseases and International Health, University of Virginia, Charlottesville; 6 Division of Infectious Diseases, University of North Carolina-Chapel Hill

**Keywords:** *Giardia*, diarrhea, growth, stunting, low-income countries

## Abstract

***Background.*** Growth stunting in children under 2 years of age in low-income countries is common. *Giardia* is a ubiquitous pathogen in this age group but studies investigating *Giardia*'s effect on both growth and diarrhea have produced conflicting results.

***Methods.*** We conducted a prospective longitudinal birth cohort study in Dhaka, Bangladesh, with monthly *Giardia* and continuous diarrheal surveillance.

***Results.*** 629 children were enrolled within the first 72 hours of life, and 445 completed 2 years of the study. 12% of children were stunted at birth with 57% stunted by 2 years. 7% of children had a *Giardia* positive surveillance stool in the first 6 months of life, whereas 74% had a positive stool by 2 years. The median time to first *Giardia* positive surveillance stool was 17 months. Presence of *Giardia* in a monthly surveillance stool within the first 6 months of life decreased length-for-age Z score at 2 years by 0.4 (95% confidence interval, −.80 to −.001; *P* value .05) whereas total number of *Giardia* positive months over the 2-year period of observation did not. Neither variable was associated with weight-for-age Z score at 2 years. In our model to examine predictors of diarrhea only exclusive breastfeeding was significantly associated with decreased diarrhea (*P* value <.001). Concomitant giardiasis was neither a risk factor nor protective.

***Conclusions.*** Early life *Giardia* was a risk factor for stunting at age 2 but not poor weight gain. Presence of *Giardia* neither increased nor decreased odds of acute all cause diarrhea.

## Abstract (precis)

This prospective longitudinal birth cohort demonstrated that asymptomatic giardiasis in the first 6 months of life is a risk factor for poor linear growth. Furthermore, asymptomatic giardiasis neither increases nor decreases odds of having an all cause acute diarrheal episode.

**(See the Editorial Commentary by Hanevik on pages 798–9.)**

*Giardia lamblia* (syn. *G. duodenalis/intestinalis*) is a flagellated unicellular eukaryotic protozoan endemic to both high- and low-income countries [1]. Fecal-oral ingestion of *Giardia* cysts leads to varied clinical syndromes ranging from acute or chronic diarrhea to long-term asymptomatic colonization [2, 3]. In a recent multicenter study in low- and middle-income countries, *Giardia* was the fourth most common pathogen isolated in both diarrheal and nondiarrheal surveillance stools in children younger than 1 year of age and the second most common pathogen in children aged 1–2 years [4]. Given the spectrum of clinical presentation, ascertaining the morbidity associated with giardiasis in low-income settings has been difficult. Two outcomes that are particularly unclear are the impact of endemic *Giardia* on growth and acute diarrhea in children.

Studies examining the effect of *Giardia* on childhood growth have been mixed. Several studies have shown an association between *Giardia* carriage and linear growth delay [5–9]. However, most of these studies were cross-sectional [7–9]. Several studies also found an association with *Giardia* and lower weight [6, 8–11]. There have been several investigations that found no association between anthropometric indices and giardiasis [12–15]. These inconsistencies have raised the possibility that host factors, *Giardia* assemblage, prolonged giardiasis, or early life giardiasis may influence *Giardia*'s effect on growth [16].

Attempts to answer the question of *Giardia*'s ability to cause acute or persistent diarrhea in children have also had mixed results. A recent meta-analysis concluded that *Giardia* generally does not cause acute diarrhea in children from low-income countries but is associated with persistent diarrhea [2]. Several investigations have claimed that *Giardia* is actually protective from acute diarrhea due to other pathogens [17–19]. Two of these studies show decreased acute diarrhea in the months following *Giardia* infection [18, 19], and one found such an association only with Rotavirus diarrhea [17]. In contrast, other investigations found increased odds ratios (OR) of acute diarrhea with *Giardia*–Rotavirus or *Giardia*-ETEC coinfections than with either pathogen alone [20].

We hypothesized that early life infection with *Giardia* (ie, in the first 6 months of life) and more *Giardia* positive monthly surveillance stools over the first 2 years of life were independent risk factors for growth failure. This hypothesis was based on recent studies showing that stunted children generally show the first signs of a downward trend in growth within the first 6 months of life [21]. We also hypothesized that infection with *Giardia* would be protective against all-cause diarrhea during the period of active giardiasis, regardless of pathogenic cause of the diarrhea as it is possible *Giardia* stimulates innate immunity that provides bystander protection from other enteric infections [22].

## PATIENTS AND METHODS

We conducted a prospective longitudinal birth cohort in an urban neighborhood of Dhaka, Bangladesh. 629 children were enrolled within 72 hours of birth from January 2008 to December 2012. We analyzed only children that completed at least 2 years of follow-up and limited our analysis to the first 2 years of life. Previous analysis of the first 147 children enrolled in this cohort has been published and included analysis of birth anthropometry as a predictor of subsequent giardiasis throughout the first year of life. No association was found [23].

The study was conducted in the urban slum of Mirpur, Dhaka. The majority of construction is mud-brick, and open sewers flow throughout the neighborhood. Our participants tended to come from the lower socioeconomic strata of Mirpur due to the area in which recruitment occurred and the location of our study clinic.

Subjects were followed by field research assistants who visited the home of each subject 3 times per week. Through these visits, data on duration of exclusive and partial breastfeeding was achieved. Close monitoring for diarrheal illness was also a priority. In the event of a diarrheal episode (defined as ≥3 unformed stools in a 24-hour window and separated from a previous diarrheal episode by at least 3 days), stool was collected by the field research assistant. Children also had nondiarrheal “surveillance” stool collected monthly and their anthropometric indices measured every 3 months. Field research assistants were trained in pediatric anthropometry measurement. Z-scores were calculated using World Health Organization growth curves. Information on socioeconomic status and education was collected via survey at enrollment. Evaluation of *Giardia* in stool samples was done via enzyme-linked immunosorbent assay (ELISA) (TechLab, Blacksburg, Virginia).

We constructed 2 multivariable linear regression models with length-for-age Z score (LAZ) and weight-for-age Z score (WAZ) at 2 years of age as the outcomes. Predictors were chosen based on previous literature around growth faltering in low-income children but included both total number of *Giardia* positive surveillance stools in the child's 2 years of life as well as presence of at least 1 *Giardia* positive surveillance stool in the first 6 months of life. Duration of exclusive breastfeeding (defined as a diet of only breast milk without formula, animal milk, water, or any other liquid/solid consumable) was also included. As all children were still partially breastfed at 2 years of age, this variable was not included in any analyses. Based on the results of these regressions, we constructed predictive models of any *Giardia* variable that was statistically significant. Predictors of giardiasis were chosen based on sanitation practices and socioeconomic factors. We utilized conditional backward stepwise selection to isolate significant predictors. A Hosmer-Lemeshow statistic was produced for our final model to assure stability [24]. A nonsignificant χ^2^ statistic was produced assuring goodness of fit.

For our secondary analysis of *Giardia*'s effect on the risk of an all-cause diarrheal episode we used generalized estimating equations to fit a binary logistic regression model, where the outcome was the presence of at least 1 diarrheal episode in each month of life, and predictors included risk factors for diarrheal disease as well as the presence of *Giardia* in surveillance stool (a time-varying predictor).

For statistical analysis, only subjects with complete data sets for all variables in a given analysis were used for that particular model. GraphPad Prism version 5.01 (GraphPad Software, Inc., La Jolla, California) was used for survival analyses. SAS version 9.4 (SAS Institute Inc., Cary, North Carolina) was used for all other statistical computation.

This study was approved by the Ethics and Research Review Committees at The International Centre for Diarrhoeal Disease Research, Bangladesh, and by the Institutional Review Board at the University of Virginia.

## RESULTS

**Figure 1.**
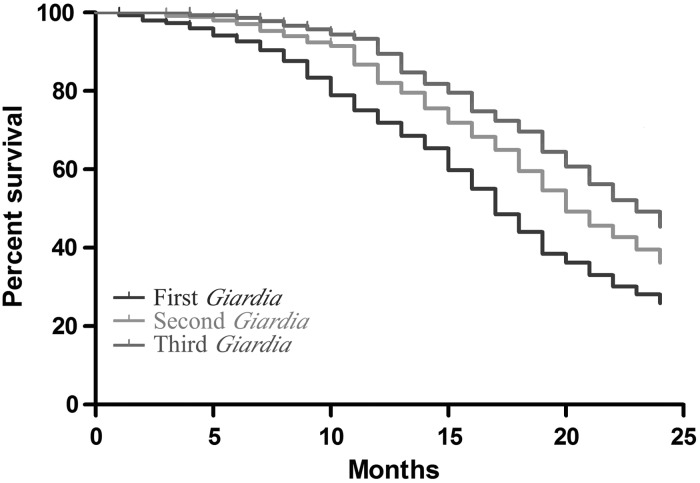
Survival plots of presence of *Giardia* in monthly surveillance stool over the first 2 years of life in urban Bangladeshi infants. *Giardia* was ubiquitous in children in Dhaka, Bangladesh, with the median time to first *Giardia* positive surveillance stool at 17 months. For second and third positive surveillance stools the median survival time was 1 and 2 months after initial infection, respectively. 7% of children had a *Giardia* positive surveillance stool in the first 6 months of life. By 2 years of age 74% of our cohort had at least 1 *Giardia* positive surveillance stool. 64% had at least 2 positive monthly stools, and 55% had at least 3.

629 children were enrolled in the cohort. 445 children completed 2 years of the study and had a surveillance stool collected for all 24 months. Enrollment characteristics did not differ between the original cohort and the 445 patients completing the study (Table 1). Children in our cohort had an average of 3.6 *Giardia* positive monthly surveillance stools throughout the first 2 years of life. The median time to first *Giardia* positive surveillance stool was 17 months. For second and third positive surveillance stools the median survival time was 1 and 2 months after initial infection, respectively (Figure 1). 7% of children had a *Giardia* positive surveillance stool in the first 6 months of life. By 2 years of age 74% of our cohort had at least 1 *Giardia* positive surveillance stool. 64% had at least 2 positive monthly stools, and 55% had at least 3.

**Table 1.** Enrollment Characteristics

Parameter	629 Children Enrolled	445 Children Completing 2 y of Study	*P* Value
Weight	2718.5 ± 411.2	2724.8 ± 418.5	.84^a^
Length	48.4 ± 2.1	48.4 ± 2.2	.67^a^
Head circumference	33.9 ± 1.4	33.9 ± 1.4	.82^a^
Low birth weight	202 (32%)	145 (32%)	.90^b^
Estimated gestational age	38.0 ± 0.7	38.0 ± 0.7	.32^a^
Male	332 (53%)	245 (55%)	.46^b^
Birth outside of a hospital setting	397 (63%)	274 (62%)	.65^b^
Any formal maternal education	399 (63%)	280 (63%)	.95^b^
Years of maternal education	3.7 ± 3.3	3.7 ± 3.4	.90^a^
Income (taka)^c^	6903.8 ± 3500.3	6920.9 ± 3249.6	.65^a^

Data displayed as average ± standard deviation for continuous variables or count (percentage) for dichotomous variables.

^a^ Mann–Whitney U Test.

^b^ Exact Pearson χ^2^ test.

^c^ 1 US Dollar = 67 to 81 Taka during the study period.

354 children had complete data sets for anthropometry and were used for LAZ and WAZ regressions. Children in this cohort had an average decline in LAZ score of 1.35 from enrollment to 2 years of age. LAZ at enrollment, income, maternal education, and early life giardiasis were statistically significant predictors of LAZ at 2 years of age. A 1-unit increase in LAZ at enrollment increased LAZ at 2 years by 0.40 (confidence interval [CI], .26–.54; *P* value <.0001). For every 1000 taka of family income LAZ at 2 years increased by 0.08 (CI, .05–.11; *P* value <.0001). Presence of any formal education in mothers increased LAZ at 2 years by 0.28 (CI, .07–.49; *P* value .009). Presence of a least 1 *Giardia* positive surveillance stool in the first 6 months of life decreased LAZ at 2 years of age by 0.4 (CI, −.80 to −.001; *P* value .05), whereas the total number of *Giardia* positive surveillance stools in the child's first 2 years of life was not a statistically significant predictor. WAZ at enrollment, length of exclusive breastfeeding, and gender were not statistically significant predictors of LAZ at 2 years.

We conducted a similar regression for change in WAZ from enrollment to 2 years of age. Children in our cohort had an average decline in WAZ of 0.5 from enrollment to 2 years of age. LAZ at enrollment, WAZ at enrollment, income, and maternal education were predictors of WAZ at 2 years. For a 1-unit increase in LAZ at enrollment, WAZ at 2 years increased by 0.17 (CI, .03–.30; *P* value .02). A 1-unit increase in enrollment WAZ increased WAZ at 2 years by 0.22 (CI, .07–.37; *P* value .003). 1000 taka of family income equated to a 0.05 increase (CI, .02–.08; *P* value .003), and presence of any formal maternal education resulted in a 0.36 increase (CI, .16–.56; *P* value .0005). Notably, neither presence of giardiasis in the first 6 months of life nor total number of *Giardia* positive surveillance stools in the child's life were statistically significant predictors of WAZ at 2 years of age. Length of exclusive breastfeeding and gender were also not statistically significant (Table 2).

**Table 2.** Prediction of Length-for-Age Z Score and Weight-for-Age Z Score at Two Years of Age in Multivariable Models

	LAZ at 2 y^a^		WAZ at 2 y^b^	
Variable	Increase in LAZ at 2 y (95% Confidence Interval)	*P* Value	Increase in WAZ at 2 y (95% Confidence Interval)	*P* Value
WAZ at enrollment	0.04 (−.11, .18)	.65	0.22 (.07, .37)	.003
LAZ at enrollment	0.40 (.26, .54)	<.0001	0.17 (.03, .30)	.02
Income (1000 taka)^c^	0.08 (.05, .11)	<.0001	0.05 (.02, .08)	.003
Exclusive breast feeding (100 d)	0.04 (−.11, .18)	.63	−0.05 (−.19, .10)	.53
Male	0.04 (−.16, .24)	.68	0.09 (−.11, .28)	.38
Any formal maternal education	0.28 (.07, .49)	.009	0.36 (.16, .56)	.0005
Presence of at least 1 *Giardia* positive month in the first 6 months of life	−0.40 (−.80, −.001)	.05	−0.20 (−.59, .18)	.30
Total number of *Giardia* positive months in the first 2 years of life	0.003 (−.026, .032)	.83	0.007 (−.02, .04)	.64

n = 354.

Abbreviations: LAZ, length-for-age Z score; WAZ, weight-for-age Z score.

^a^ R^2^ = 0.26.

^b^ R^2^ = 0.20.

^c^ 1 US Dollar = 67–81 Taka during the study period.

Complete data on 391 patients at 6 months of age were available to investigate risk factors for early life *Giardia* (defined as at least 1 *Giardia* positive surveillance stool in the first 6 months of life)*.* Predictors entered into our original model included sex, income, presence of any maternal education, months of exclusive breastfeeding (limited to the first 6 months), presence of an animal in the home, presence of an open drain/sewer outside the bedroom, presence of an open drain/sewer outside the kitchen, routine covering of drinking water vessels, routine hand washing by primary caregiver prior to feeding the child, use of piped drinking water, use of a tube well for drinking water, presence of a toilet or septic tank, use of a pit latrine, and use of open defecation. After selection, only months of exclusive breastfeeding (OR: 0.72, CI, .58–.90; *P* value .004) and caregiver hand washing prior to feeding the child (OR: 0.33, CI, .11–.97; *P* value .04) remained as statistically significant predictors early life *Giardia* (data not shown). A Hosmer-Lemeshow statistic was produced for our final model with nonsignificant χ^2^ statistic of 12.25 (*P* value .09).

Finally, we investigated the association between *Giardia* carriage and all-cause diarrhea. Variables included in our model to predict presence of at least 1 diarrheal episode in a given month included any maternal education, income, exclusive breastfeeding, WAZ at enrollment, LAZ at enrollment, sex, and presence of *Giardia* in surveillance stool. 274 children had complete data and were analyzed with this model. In these children, 1164 of the total 6576 child-months (17.70%) were positive for a diarrheal episode with an average of 6.07 episodes of diarrhea per child over the first 2 years of life. Of those diarrhea positive child-months, 36.25% (422 child-months) had *Giardia* detected in the surveillance stool. Only exclusive breastfeeding was significantly associated with decreased diarrhea (*P* value <.001). No variables in our model were associated with increased diarrhea. Importantly, presence of *Giardia* in the child's surveillance stool was neither protective nor a risk factor for that child having an acute diarrheal episode.

We then repeated this analysis but separated presence of Giardia into 2 separate covariates; first incidence of *Giardia* detected in the surveillance stool and subsequent incidence of *Giardia* detected in the surveillance stool. This did not change the model with exclusive breastfeeding remaining significant (*P* value <.001) and neither of the *Giardia* covariates being associated with diarrhea (data not shown).

## DISCUSSION

Our study demonstrated that giardiasis in the first 6 months of life but not the total number of *Giardia* positive surveillance stools over the first 2 years of life, was inversely associated with linear growth. Longitudinal analysis of growth in low-income countries has shown the first 6 months of life to be a crucial window that determines either healthy or diminished height gain [21, 25]. *Giardia* has been shown to disrupt intestinal tight junctions in vitro and increased intestinal permeability early in life is associated with growth stunting [23, 26]. Giardiasis in weanling mice disrupts the villus:crypt architecture in the presence of chronic inflammation and impairs growth [27]. Furthermore, although some *Giardia* strains are known to cause intestinal inflammation, it has been shown that the levels of inflammation decrease with subsequent infections [28, 29]. This implies that there is acquired protection against severity of giardiasis but not from reinfection, which may be the reason having a first *Giardia* infection in early life influences growth more than prolonged infection.

Our findings are significant as they potentially explain the discrepancy in the literature investigating *Giardia*'s effect on growth. Cross-sectional studies that identified associations between *Giardia* and decreased length likely represented identification of children who had predisposing risk factors to giardiasis and may have had early exposure. Likewise, studies that found no such association may have not discriminated between early giardiasis and later infection.

We showed hand washing practices and exclusive breastfeeding were inversely associated with early life giardiasis, which is consistent with several studies showing similar protection [30, 31]. This suggests that caretakers are transmitting *Giardia* from the environment or fecal-oral route on their hands. Daycare outbreaks of *Giardia* in the United States support this concept, thus suggesting that epidemic and endemic routes of transmission may be the same [32, 33]. There is also literature that suggests that breast milk protects against symptomatic *Giardia* via secretory IgA although offering no protection against colonization or reinfection [34]. Contradictory work shows anti-*Giardia* IgA offers no protection against diarrheal or asymptomatic infection nor occurrence of repeat infections [35]. This may be explained by work showing breast milk to be protective against only the first infection of *Giardia* but not chronic colonization [36]. Given our findings that early life giardiasis was a risk factor for growth stunting, it seems that maintenance of exclusive breastfeeding through 6 months of age and good hand hygiene may be crucial interventions in protection from *Giardia* associated stunting.

Our results also indicate that presence of *Giardia* carriage is neither a risk factor for acute diarrheal illness nor is it protective. This corroborates the 2 longitudinal studies in the literature that found no change in incidence of acute diarrhea during *Giardia* positive periods [14, 17]. The hypothesis that *Giardia* may be protective against other enteropathogens comes from studies comparing diarrheal and nondiarrheal surveillance stools that found lower incidence of *Giardia* in diarrheal stools. However, we postulate an alternative explanation that colonizing *Giardia* is being washed out of feces during acute diarrheal episodes from other pathogens and thus does not necessarily imply *Giardia* is protective.

This study has several important limitations. First, although ELISA has both high sensitivity and specificity, it may be inferior to polymerase chain reaction (PCR), and thus we may have missed low-level *Giardia* colonization. Second, we did not differentiate *Giardia* assemblages. It has been shown that although assemblage B is more prevalent in Bangladesh, assemblage A had a stronger association with diarrhea [37]. Associations between assemblages A and B with symptoms are inconsistent across both experimental and field studies [16]. Thus, it may be that a particular assemblage is more or less associated with our outcomes of interest and that the signal is lost when the summative burden of all *Giardia* is investigated. Third, the confidence interval for *Giardia*'s association with poor linear growth is large, making accurate estimation of the effect size difficult. This is likely due to the low number of children who had early life giardiasis. A higher sample size would have made this estimation more accurate. Lastly, information regarding coinfections, intestinal microbiota, and markers of permeability and intestinal inflammation was beyond the scope of the present study but likely is very relevant to the primary outcomes of growth and diarrhea and may interact with asymptomatic giardiasis.

The study has particular strengths that are unique to other outcome-based studies of pediatric endemic giardiasis. First, intensive home-based surveillance ensured that diarrheal surveillance and breastfeeding data were accurate. Second, while ELISA may be inferior to PCR, it is superior to microscopy, which many of the older studies of giardiasis relied upon. Third, the longitudinal design incorporating surveillance and diarrheal stools allowed us to investigate the incidence of diarrhea during periods of known *Giardia* positivity. Lastly, our study had the benefit of continued surveillance in a birth cohort allowing us to detect early life *Giardia* and analyze this as a separate risk factor.

## CONCLUSIONS

*Giardia* was common in our cohort. Using frequent sampling in a prospective pediatric cohort enrolled at birth, we have helped clarify important considerations in resolving the endemic pediatric giardiasis conundrum. For the first time, we show that infection with *Giardia* within the first 6 months of life was an independent risk factor for linear growth failure but not poor weight gain, whereas total number of *Giardia* positive monthly surveillance stools had no effect on growth. Presence of *Giardia* had no effect on the likelihood of a child developing acute diarrhea. In this regard, efforts to prevent *Giardia*-associated growth sequelae may have the greatest impact during the first 6 months of life. Our data support that these measures focus on introduction of hand-washing practices and exclusive breastfeeding. Whether screening and treatment of giardiasis in early life could salvage later growth delays is an important question for future study. Further investigation into the effects of specific *Giardia* assemblages and interactions with co-pathogens may provide further understanding of the subacute effects of this ubiquitous but enigmatic pathogen.
